# ﻿Description of two new species of the genus *Tillicera* Spinola (Coleoptera, Cleridae, Clerinae), with new synonyms, new distributional records, and an updated key

**DOI:** 10.3897/zookeys.1095.80097

**Published:** 2022-04-13

**Authors:** Hiroyuki Murakami, Roland Gerstmeier, Kaoru Sakai

**Affiliations:** 1 Entomological Laboratory, Faculty of Agriculture, Ehime University, 3-5-7 Tarumi, Matsuyama, 790-8566 Japan Ehime University Matsuyama Japan; 2 Zoologische Staatssammlung München, Münchhausenstraße 21, 81247 München, Germany Zoologische Staatssammlung München München Germany; 3 Ôta-ku, Tokyo, Japan Unaffiliated Ôta-ku Japan

**Keywords:** Antennal sensilla, checkered beetles, Oriental Region, taxonomy

## Abstract

Two species, *Tillicerafortis***sp. nov.** and *Tilliceraspinosa***sp. nov.**, are newly described. New distributional records are presented for *Tilliceracallosa* Gerstmeier & Bernhard, 2010, *Tillicerajavana* Spinola, 1844, *Tillicerapseudocleroides* Gerstmeier & Bernhard, 2010, *Tillicerasoror* Schenkling, 1902, and *Tilliceratonkinensis* Gerstmeier & Bernhard, 2010. *Cleruspostmaculatus* Nakane, 1963 **syn. nov.** is synonymized with *Tilliceraihlei* Corporaal, 1949. The presence of sensory organs (sensilla) on the ventral surface of the antennae is discovered in *Tillicera* and *Hemitrachys* for the first time. A key to the valid species of *Tillicera* is provided.

## ﻿Introduction

The genus *Tillicera* Spinola (Coleoptera, Cleridae) belongs to the subtribe Clerina Latreille, 1802 of the tribe Clerini Latreille, 1802 (Bartlett 2021). This genus forms part of the *Tillicera* genus group with *Apopempsis* Schenkling, 1903, *Cardiostichus* Quedenfeldt, 1885, *Hemitrachys* Gorham, 1876, *Placocerus* Klug, 1837, and *Plathanocera* Schenkling, 1902 (Gerstmeier and Stapel 2016). Until now, 17 species of the genus have been known from the Oriental region (Gerstmeier and Bernhard 2010; Yang et al. 2011). Many species are known for their mimicry of the wasp family Mutillidae (Shelford 1902; Opitz 2010).

In this paper, we describe two new species and synonymize one species, *Cleruspostmaculatus* Nakane, 1963a with *Tilliceraihlei* Corporaal, 1949. We provide an updated key to the valid species of the genus, plus photographs illustrating the antennal sensory organs previously discussed by Yang et al. (2011).

## ﻿Materials and methods

The dissection method of terminal parts and genitalia follows Murakami (2016). The terminology of male genitalia follows Bartlett (2021). The material in this study is deposited in following institutions and private collections:

**EUMJ**Ehime University Museum, Matsuyama, Japan;

**KSCJ** private collection of Kaoru Sakai, Tokyo, Japan;

**MMCJ** private collection of Masafumi Matsumura, Okinawa, Japan;

**MTCJ** private collection of Minoru Tanaka, Japan;

**NHMB**Natural History Museum Basel, Switzerland;

**NHMI** Natural History Museum and Institute, Chiba, Japan;

**NMPC**National Museum Prague, Czech Republic;

**OLML**Oberöstereichisches Landesmuseum Linz, Austria;

**QSBG**Queen Sirikit Botanic Garden, Entomology Section, Chiang Mai Prov., Thailand;

**RGCM** Roland Gerstmeier Collection, Munich (deposited in ZSM, Zoological State Collection Munich), Germany;

**SEHU**Systematic Entomology, Hokkaido University, Sapporo, Japan.

The original spelling of label data is indicated by double quotation marks (“ ”); line brakes are indicated by a slash (/).

The abbreviations for measurements are as follows:

**BL** body length (from tip of head to elytral apex);

**EL** elytral length (from basal margin to apex along suture);

**EW** maximum conjoined width of elytra;

**EyD** distance between eyes in dorsal view;

**EyW** maximum width of a single eye in dorsal view;

**PL** maximum length of pronotum;

**PW** maximum width of pronotum.

## ﻿Taxonomic account

### ﻿Genus Tillicera

Taxon classificationAnimaliaColeopteraCleridae

Spinola, 1841

19EECDD3-1A4F-5BC6-B9EC-44DF1DA1C27A

Tillicera Spinola, 1841: 73; Gerstmeier and Bernhard 2010: 3; Gerstmeier and Stapel 2016: 539.

#### Diagnosis.

The genus *Tillicera* is closely related to *Hemitrachys* Gorham but differs from it by the following characteristics (Yang and Yang 2013; Gerstmeier and Stapel 2016): antennomeres V–XI not clubbed; elytral length to width ratio 2.01–2.37; pulvillar formula 4–4–2, 4–4–3 or 4–4–4 (*Hemitrachys*: antennomeres V–XI clubbed; elytral length to width ratio 1.58–1.80; pulvillar formula 4–2–2).

#### Remarks.

In male and female adults of *Thanasimussubstriatus*, sensilla basiconica were distributed in clusters that formed a line along the posterior border from the fifth to the eighth antennomere (Zhang et al. 2021). The area of the apical margin of antennae in ventral view, which is vested with sensilla basiconica, was discovered in *Tilliceracallosa*, *Tillicerafortis* sp. nov., *Tilliceraspinosa* sp. nov., and *Hemitrachystubericollis* Yang & Yang, 2013 for the first time (Figs 29–31, 34, 35). The genus *Tillicera* is included in the *Tillicera* genus group with *Apopempsis*, *Cardiostichus*, *Hemitrachys*, *Placocerus*, and *Plathanocera* (Gerstmeier and Stapel 2016); the monophyly of the genus group, however, is questioned by Bartlett (2021).

In the first author’s observations, this remarkable antennal structure is also seen in two other genera, *Clerus* Geoffroy, 1762 and *Omadius* Laporte, 1838, which are included into the subtribe Clerina Latreille, 1802 (see Bartlett 2021). Further investigation is required to clarify the intergeneric relationships.

### ﻿ Tillicera callosa

Taxon classificationAnimaliaColeopteraCleridae

Gerstmeier & Bernhard, 2010

2BC8620B-098E-5B81-B13E-4CE8F5FD52D0

Figs 4 , 31 , 36–40

Tillicera callosa Gerstmeier & Bernhard, 2010: 14, figs 4, 25. Type locality India, Darjeeling District.

#### Specimens examined.

**Vietnam**: Mt. Tam Dao, Vinh Phuc Prov., 10.V.1996 (KSCJ, 2 males).

#### Additional description.

**Male.** Antennomeres V–X with an area vested with sensilla basiconica at apical margin in ventral view; XI without pit-like sensillum. Elytra with two large callous areas at base. Tarsal pulvillar formula 4–4–2; protarsomeres I and II with large lobed pulvilli; III and IV with large bilobed pulvilli; mesotarsomers I with vestigial minute pulvillus; II with small lobed pulvillus; III and IV with small bilobed pulvilli; metatarsomeres I and II without pulvilli; III and IV with small bilobed pulvilli.

Abdominal sternite V almost transverse. Pygidium weakly emarginated at apical margin (Fig. 36); apical margin of ventrite VI (Fig. 37) emarginated at middle; spicular forked, long (Fig. 38).

Tegmen (Fig. 39a–c) with dorsal and ventral sinuses at apical 1/6 of total length; tegminal arms long, extending 1/3–2/3 of total length. Median lobe (Fig. 40a–c) longer than tegmen; plate simple, without denticles.

#### Remarks.

This species was originally described based on a single female specimen from India. In this paper, we describe the male in detail. This species is also related to *Hemitrachysbizonatus* Gorham, 1876 based on the structure of the male genitalia.

#### Distribution.

India. New record: Vietnam.

### ﻿ Tillicera ihlei

Taxon classificationAnimaliaColeopteraCleridae

Corporaal, 1949

DC0B3A2E-1DE2-58BB-B177-D67A33EB8480

Figs 9 , 10

Tillicera ihlei Corporaal, 1949: 99 (type locality Indonesia, Java); Gerstmeier and Bernhard 2010: 19, figs 9, 30–31
Clerus postmaculatus Nakane, 1963a: 46 (type locality Japan, Nakanoshima Is.); 1963b: 183; Miyatake 1985: 156. syn. nov.

#### Type specimen examined.

***Holotype*** of *Cleruspostmaculatus*. **Japan**: “NAKANOSHIMA/ Is. Tokara/ 7.vii.1960/ M. Sato leg.” (SEHU, 1 male).

#### Other specimens examined.

**Japan**: [Kagoshima] Kankake-dake, alt. 220 m, Yakushima, 19.VII.–22.VII.2006, T. Yamauchi leg. (EUMJ, 3 exs); same locality and collector, 8.VI.–28.VI.2007, (EUMJ, 2 exs); same locality and collector, 28.VI.–30.VII.2007, (EUMJ, 5 exs); Hanyama, alt. 250 m, Yakushima, 22.VI.–22.VIII.2006, T. Yamauchi leg. (EUMJ, 3 exs, Malaise Trap); same locality and collector, 28.VI.–30.VII.2007, (EUMJ, 3 exs); same locality and collector, 27.VII.–30.VII.2007 (EUMJ, 3 exs); Aiko-dake, alt. 150 m, 28.VI.–29.VII.2007, Y. Takeo leg. (EUMJ, 1 ex., Malaise Trap); Miyanoura, Yaku-shima, 10.VII.1961, K. Ueda leg. (EUMJ, 1 female); Ikari, Amami-Oshima, 19.VI.1961, T. Shibata leg. (EUMJ, 1 male); same locality and collector, but 3.VII.1961. (EUMJ, 1 male); Mt. Otake, Toshima-mura, Nakano-shima, 23.VI.2004, J. Yamasako leg. (EUMJ, 1 female); Toshima, Nakano-shima, 1–7.VII.2009, M. Matsumura leg. (MMCJ, 1 ex.). [Okinawa] Iji, Kunigami-son, Okinawa-jima, 2.V.1993, M. Matsumura leg. (MMCJ, 1 male); Okuni-rindo, Kunigami-son, Okinawa-jima, 13.VI.1997, K. Inada leg. (KSCJ, 1 male); Aha, Kunigami-son, Okinawa-jima, 24.V.2012, M. Matsumura leg. (MMCJ, 1 female). **Taiwan**: [Miaoli] Xuejian Recreation Area, Tainan Township, 10. VI. 2015, S. Shih leg. (EUMJ, 8 males & 18 exs) [Taitung] 9.VI.1971, Y. Maeda leg. (EUMJ, 1 female); Jinping-forestroad, Chihshang Township, 19.VI.2011, J. Yamasako leg. (EUMJ, 1 male). [Kaohsiung] Liukei Dist., 29.IV-8.V.1982, H. Takizawa leg. (KSCJ, 1 ex.); Tengjhih, Taoyuan Dist., 14.V.1991, W. L. Chen leg. (NHMI, 1 female). [Nantou] Nanzankei, Puli Township, 8.V.1965, T. Shirozu leg. (EUMJ, 1 male); same locality, 6.V.1971, Y. Hayashi leg. (EUMJ, 1 female); same locality, 17.IV.1977, W. Suzuki leg. (KSCJ, 1ex.); Sung Kang, Renai Township, 8.VIII.1983, K. Ra leg. (KSCJ, 1ex.); Gaofeng, alt. c. 1700 m, Musha, Renai Township, 24–28.VIII.2016, M. Tanaka leg. (MTCJ, 1 male); Mt. Rozan, alt. c. 1200 m, Renai Township, 29–30.VII.1976, H. Kan leg. (EUMJ, 1 female); Napankanshan, Renai Township, alt. 2050–2800 m, 20.V.1991, A. Saito leg. (NHMI, 1 male, 1 female). **Laos**: Mt. Phu Pan, Ban Saleui, Xam Neua, Houa Pan Prov., 9.V.2007, T. Mizusawa leg. (KSCJ, 1 male); Mt. Phou Pan, Ban Saleui, Hua Phan Prov., 20°13'30"N, 103°59'26"E, 1350–1900 m, 22.V.2011, C. Holzschuh & Native leg. (OLML, 1 ex.). **Thailand**: Doi Pui, Chiang Mai Prov., 28.IV.1983, H. Kan leg. (EUMJ, 1 ex.); Meo Village, Chiang Mai Prov., 1.V.1987, S. Saito leg. (KSCJ, 1ex.); Chang Dao, Pa kia, Chiang Mai Prov., 4–5.V.2000, K. Okajima leg. (KSCJ, 1 ex.); Wiang Pa Pao, Chiang Rai Prov., 17–21.V.2015, K. Takahashi leg. (KSCJ, 1 ex.); same locality, 20–29.V.2017, K. Takahashi leg. (KSCJ, 3 exs); Chiang Rai, Wiang Pa Pao, Mae Chedi, alt. 1200 m, 9–12.V.2018, S. Imada & S. Inoue leg. (EUMJ, 2 exs); Mae Hong Son, Ban Huai Po, 1600–2000 m, 9.–16.V.1991, J. Horah leg. (NMPC, 1 male). **Myanmar**: Maymyo (Pin Oo Lwin), alt. c. 1000 m, Mandalay Div., 6.VII.2001, Y. Kusakabe leg. (KSCJ, 1 ex.).

**Figures 1–12.**
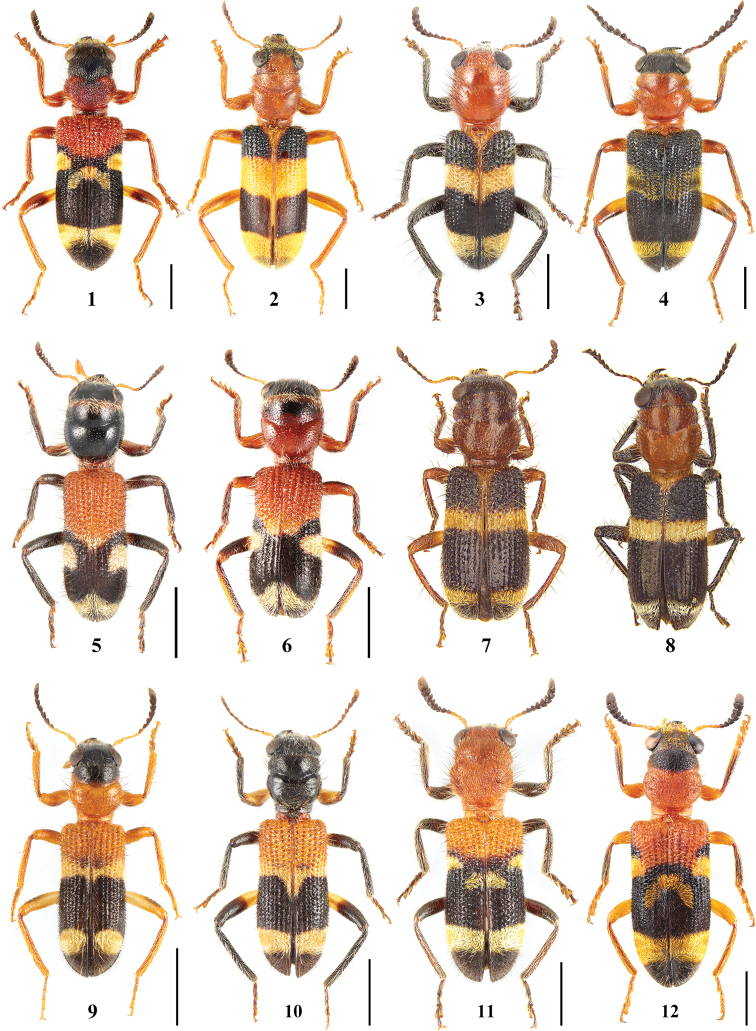
Habitus of *Tillicera* species **1***Tilliceraauratofasciata***2***T.aurivillosa***3***T.bibalteata***4***T.callosa***5, 6***T.cleroides***7, 8***T.hirsuta***9, 10***T.ihlei***11, 12***T.javana*. Scale bars 2 mm.

#### Description.

For more details see Gerstmeier and Bernhard (2010). The color variation is recognized as follows (Figs 9, 10): pronotum black at apical 1/3 and reddish at basal 2/3; black at apical 1/2 and reddish on remainder, wholly black and partly reddish at base; elytra sometimes partly black between basal reddish area and basal whitish fascia; femora yellow at base and black at apex; however, that color area is variable. Antennomeres V–X with a smooth area at apical margins in ventral view; XI without pit-like sensillum.

#### Remarks.

*Cleruspostmaculatus* was described from Nakano-shima Is., Okinawa Pref., Japan, by Nakane (1963a). After close examination of the type specimen, we conclude its synonymy with *Tilliceraihlei*.

#### Distribution.

Indonesia. New records: Japan, Laos, Myanmar, Taiwan, Thailand.

### ﻿ Tillicera javana

Taxon classificationAnimaliaColeopteraCleridae

Spinola, 1844

7211F97A-D670-5CA0-A864-2175F783E71D

Figs 11 , 12

Tillicera javana Spinola, 1844: 160, pl 12, fig. 2 (type locality Indonesia, Java); Schenkling 1906: 262; Gerstmeier and Bernhard 2010: 21.
Tillicera javanica : Desmarest 1860: 241, fig. 169; Gorham 1893: 567.
Tillicera javana ab. luchti Corporaal, 1949: 100 (type locality Indonesia, Sumatra); Gerstmeier and Bernhard 2010: 23.

#### Specimens examined.

**Laos**: Phou Samsoum, Xiang Khouang Prov., 2.VI.2007, J. Yamasako leg. (EUMJ, 1 ex.); Souy Dist., West of Phonsavan 40–50 km, Xiang Khouang Prov., 17–18.VI.2008, J. Yamasako leg. (EUMJ, 1 female); Mt. Phu Pan, Ban Saleui, Xam Neua, Houa Pan Prov., alt. 1500–1700 m, 4.V.2002, N. Ohbayashi leg. (EUMJ, 1 ex.); same locality, alt. 1600–1900 m, 2–10.IV.2005, unknown collector (EUMJ, 1 ex.); same locality, alt. 1700–1800 m, 17–20.VI.2003, N. Ohbayashi leg. (EUMJ, 2 exs); same locality, alt. 1750 m, 16–20.V.2004, M. Sato leg. (EUMJ, 1 ex.); same locality, alt. 1400–1500 m, 20–24.VI.2003, N. Ohbayashi leg. (EUMJ, 1 ex.). **Thailand**: Khao Yai, 10. VI.1992. (EUMJ, 9 exs); Doi Pui, Chiang Mai Prov., 8.V.1983, H. Kan leg. (EUMJ, 1 ex.). **Vietnam**: Mt. Tam Dao, alt. 930 m, Vinh Phuc Prov., 1–7.V.1996, Y. Arita leg. (EUMJ, 4 exs); Mt. Lang Biang, alt. c. 1800 m, Lac Duong Dist., Lam Dong Prov., 31.V.2016, M. Matsumura leg. (EUMJ, 1 female).

**Figures 13–22.**
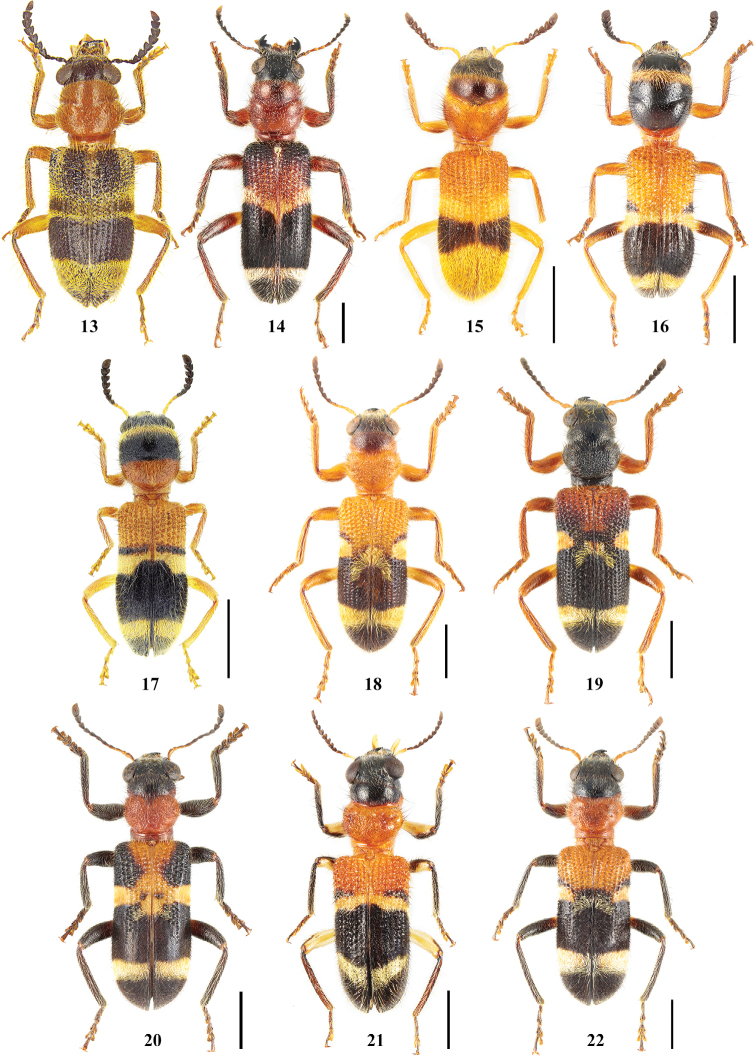
Habitus of *Tillicera* species **13***Tilliceramichaeli***14***T.obscura***15***T.paula***16, 17***T.pseudocleroides***18, 19***T.sensibilis***20***T.soror***21***T.tonkinensis***22***T.wenii*. Scale bars 2 mm.

#### Additional description.

This species is variable in its pronotal color (Gerstmeier and Bernhard 2010). Additional variation is recognized as follows: head and pronotum completely reddish (Fig. 11); legs yellowish (Fig. 12). Antennomeres V–X vestigial with an area vested with sensilla basiconica at apical margin in ventral view; male XI of dorsal surface with pit-like sensillum.

#### Remarks.

Opitz’s (2010, fig. 217) photograph labelled *T.javana*, used to illustrate a likeness to mutilid wasps, appears to be a misidentified specimen of *Tilliceratonkinensis* Gerstmeier & Bernhard, 2010

#### Distribution.

Indonesia (Java, Sumatra), Malaysia (Borneo, Sabah). New records: Laos, Thailand, Vietnam.

### ﻿ Tillicera pseudocleroides

Taxon classificationAnimaliaColeopteraCleridae

Gerstmeier & Bernhard, 2010

878EC215-0FD1-5D98-BDA7-9B9309D7C1A3

Figs 16 , 17

Tillicera pseudocleroides Gerstmeier & Bernhard, 2010: 27, figs 15, 39–40. Type locality Indonesia (Java, Sumatra), Malaysia (Pahang).

#### Specimens examined.

**Laos**: Attapeu Prov., Thong Kai Ohk, Ban Kachung (Mai) env., 1200–1450 m, 15°01.02'N, 107°26.27'E, 10.-24.VI.2011, M. Brancucci, M. Geiser, D. Hauck, Z. Kraus, A. Phantala & E. Vongphachan (NHMB, 1 female); Souy Dist., West Phonsavan 40–50 km, Xieng Khouang Prov., 17–18.VI.2008, J. Yamasako leg. (EUMJ, 1 female).

#### Additional description.

The colour of the pronotum, elytra, and legs are variable in this species (Figs 16, 17). Antennomeres V–X with an area vested with sensilla basiconica at the apical margin in ventral view.

#### Distribution.

Indonesia (Java, Sumatra), Malaysia. New record: Laos.

### ﻿ Tillicera soror

Taxon classificationAnimaliaColeopteraCleridae

Schenkling, 1902

26B90252-B32F-5746-A96E-50D5DC87E6FC

Fig. 20

Tillicera soror Schenkling, 1902: 127 (type locality Bhutan); Schenkling 1903: 121, pl. 1, fig. 10; Corporaal 1939: 26; Gerstmeier and Bernhard 2010: 29, figs 16, 41, 42.
Rhytidoclerus soror : Pic 1934: 133.

#### Specimens examined.

**Vietnam**: Mt. Phang Xi Pang, N. rdg., alt. 1950–1970 m, Hoang Lien Son Mts., Lai Chau Prov., 11.V.1995, A. Saito leg. (KSCJ, 1 male); Mt. Pia Oac, Deo Kolea, alt. 1250–1300 m, Cao Bang Prov., N. Vietnam, 23–24.V.1999, A. Saito leg (KSCJ, 1 female).

#### Additional description.

Antennomeres V–X with an area vested with sensilla basiconica at apical margin in ventral view; male antennomere XI with pit-like sensillum. Tarsal pulvillar formula 4–4–2, but in one male from Vietnam 4–4–4.

#### Distribution.

Bhutan, India, Nepal. New record: Vietnam.

### ﻿ Tillicera tonkinensis

Taxon classificationAnimaliaColeopteraCleridae

Gerstmeier & Bernhard, 2010

0C1A6FA7-7165-593A-92A2-AF6CF0483C9F

Fig. 21

Tillicera tonkinensis Gerstmeier & Bernhard, 2010: 32, figs 17, 43–44. Type locality Vietnam, Tam dao.

#### Specimens examined.

**Laos**: Salavan Prov., 16°08'N, 106°42.43'E, Xe Xap NPA, c. 15 km NE of Ta-oy, BAN DOUB env., 600–900 m, 26.-30.v.2012. NHMB Basel, Expedition Laos 2012: M. Brancucci, M. Geiser, V. Phanthavong, S. Xayalath (NHMB, 1 ex.).

#### Additional description.

Antennomeres V–X with an area vested with sensilla basiconica at the apical margin in ventral view.

#### Distribution.

Vietnam. New record: Laos.

### ﻿ Tillicera fortis sp. nov.

Taxon classificationAnimaliaColeopteraCleridae

22CA2EFF-D9E4-52D9-9BF0-CABCD522BF4D

http://zoobank.org/E1E56C85-8AC4-4A6A-AA70-9A4AA6D0C692

**Figures 23–27.**
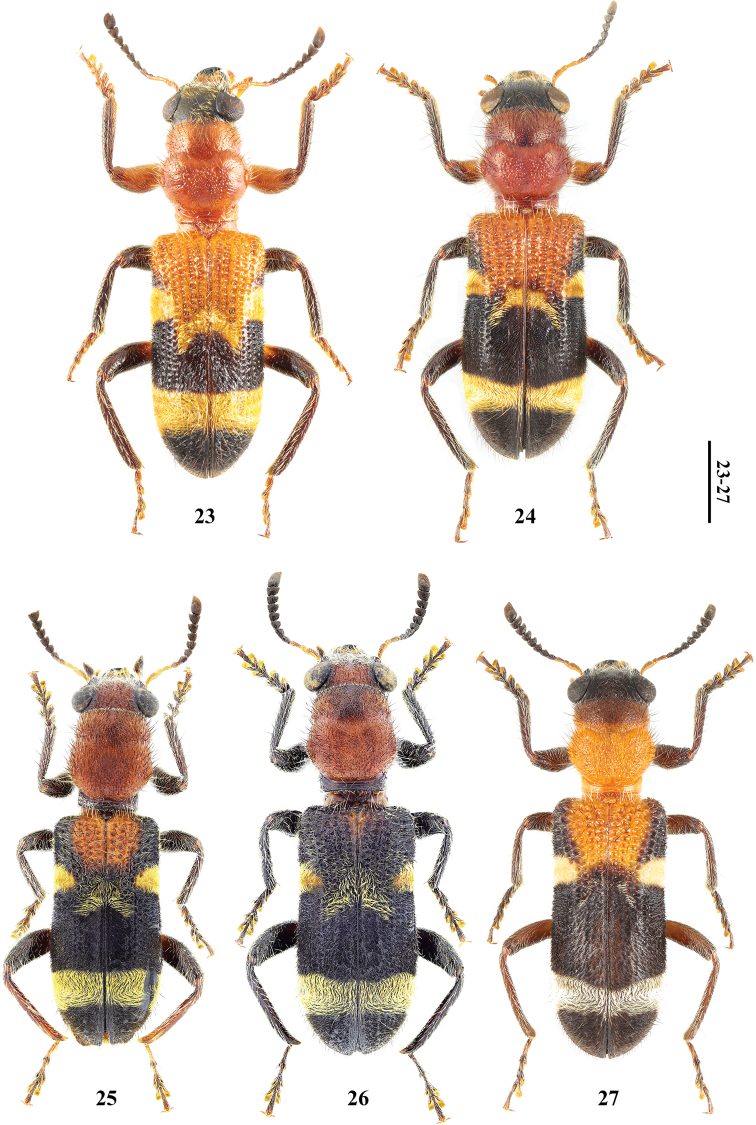
Habitus of *Tillicera***23***Tillicerafortis* sp. nov., Holotype, male **24** ditto, paratype, female **25***T.spinosa* sp. nov., Holotype, male **26** ditto, paratype, male **27** ditto, paratype, female. Scale bars 2 mm.

Figs 23 , 24 , 41–45

#### Types.

***Holotype*. Laos**: “E. Phonsavan, 25 km,/ Xiang Khouang/ 19.VI.2005/ J. Yamasako leg.”, (EUMJ, 1 male) ***Paratype.* Thailand**: “Doi Pui/ Chiang Mai Prov./ N. Thailand/ 13–14. VI. 1979/ W. Suzuki leg”, (EUMJ, 1 female).

#### Diagnosis.

This species is similar to *T.auratofasciata*, but can be distinguished by the following characteristics: male antennomere X as wide as long; punctation of pronotum sparse; tegmen with deep sinus at apical 1/4; denticles of phallic plates extending to apex (vs. *T.auratofasciata*: male antennomere X wider than long; punctation of pronotum dense; tegmen with sinus at apical 1/6; denticles of phallus not extending to apex).

#### Description.

**Male.** Head black; antennomeres V–XI, apex of profemora, meso- and metafemora except for base, tibiae, and tarsi brownish black; pronotum and profemora except for apex and base of meso- and metafemora reddish; elytra reddish at basal 1/3, remainder black, with three yellow maculations extending from lateral margin of basal 1/4 interrupted before suture, outwardly curved from suture of basal 1/3, and transversely at apical 1/4, confluent with suture.

Head including eyes slightly narrower than pronotum; labrum incised medially; maxillary terminal palpomeres digitiform; labial terminal palpomeres widely triangular; postgular plate narrow. Antennomere I claviform; II compact; III twice as long as II; IV–X triangular, becoming gradually widened; IX as long as wide; X slightly wider than long; V–X with an area vested with sensilla basiconica at apical margin in ventral view; XI with a pit-like sensillum.

**Figures 28–31.**
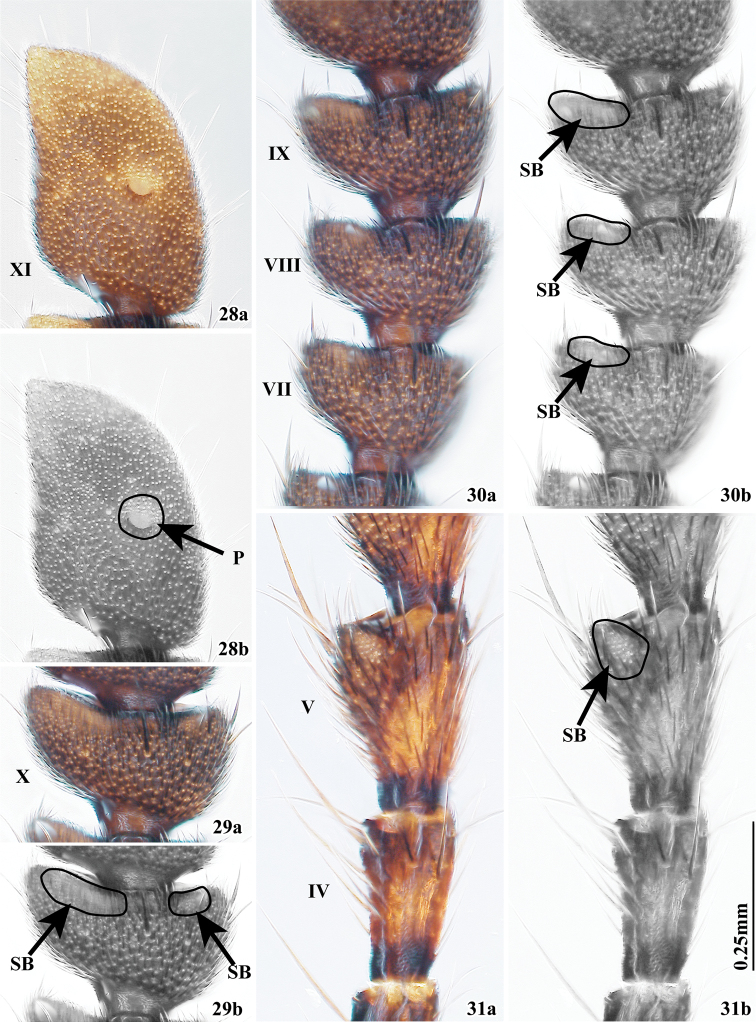
Antennae of *Tillicera* spp. (**28** dorsal view **29–31** ventral view) **28** male antennomere XI of *T.spinosa* sp. nov. **29** female antennomere X of *T.spinosa* sp. nov. **30** female antennomeres VII–IX of *T.spinosa* sp. nov. **31** male antennomeres IV and V of *T.callosa*. Abbreviations: **P** pit-like sensillum **SB** an area of sensilla basiconica.

Pronotum slightly longer than wide, widest at middle, with fine dense punctures. Mesoventrite with short anterior process.

Elytra oblong, parallel sided, covered with ten striae; striae I–III absent before basal oblique yellow fascia; IV–VI extending after middle; VII and VIII weakly punctured after base of lateral yellow fascia; IX and X obscuse.

Tibiae stout, with longitudinal carina; tibial spur formula 1–2–2; tarsal pulvillar formula 4–4–4; pro- and mesotarsomeres I and II with large lobed pulvilli; III and IV with large bilobed pulvilli; metatarsomeres I with vestigial minute pulvillus; II with small lobed pulvillus; III and IV with large bilobed pulvilli; claws with basal denticles.

Abdominal sternite V deeply, marginated at apical margin. Pygidium (Fig. 41) almost transverse at apical margin; ventrite VI (Fig. 42) broadly emarginated at apical margin; spicular fork (Fig. 43) long, without intraspicular plate.

Tegmen (Fig. 44a–c) with dorsal and ventral sinus at apical 1/4; parameroid lobes tapered posteriorly at apex in lateral view, flattened at apex; tegminal arms short, extending middle to basal 1/4 of total length. Median lobe (Fig. 45a–c) slightly shorter than tegmen; plates with rows of denticles on apex of dorsal and ventral sides, on left side in ventral view these are positioned at apical 1/5, on right side in ventral view they are very short.

**Female.** Similar to male but distinguished by the following characteristics: antennomere XI without pit-like sensillum; sternite V with shallower emargination at apical margin. Elytral striae VI to VIII weakly punctured posterior to base of lateral yellow fascia.

#### Measurements and ratios.

**Male** (*N* = 1). BL 8.80 mm; PL 2.60 mm; PW 2.25 mm; EL 6.20 mm; EW 2.80 mm; EyW 0.50 mm; EyD 0.95 mm; PL/PW 1.16; EL/EW 2.21; EL/PL 2.38; EW/PW 1.24; EyD/EyW 1.90. **Female** (*N* = 1). BL 8.65 mm; PL 2.45 mm; PW 2.25 mm; EL 6.20 mm; EW 2.90 mm; EyW 0.55 mm; EyD 1.05 mm; PL/PW 1.09; EL/EW 2.14; EL/PL 2.53; EW/PW 1.29; EyD/EyW 1.91.

#### Etymology.

This specific name is derived from the Latin *fortis* (sturdy), referring to the stout tibiae.

#### Distribution.

Laos, Thailand.

### ﻿ Tillicera spinosa sp. nov.

Taxon classificationAnimaliaColeopteraCleridae

754546A1-0C64-5726-B846-4BAF91904BCC

http://zoobank.org/2E35F6F1-8F4A-4BB9-957F-82173B1EB70E

Figs 25–27 , 28–30 , 46–50

#### Types.

***Holotype.*** “Doi Pui, 1400-/ 1500 m, Chiang Mai, N. Thailand, 19-VI-1983, T. Shimomura leg”. (RGCM, 1 male, erroneously cited in Gerstmeier and Bernhard (2010) under *T.auratofasciata*). ***Paratypes*. Laos**: “NE LAOS/ Phu Pan, 1,750 m/ Ban Saleui, Xam Neua/ Houa Phan Prov./ 16–23.VI.2003/ Shinji Nagai leg.” (KSCJ, 1 female). **Myanmar**: “near Kalaw/ 1,000–1,300 m in alt./ Shan Sta., Myanmar/ 10–25.V.2005/ Y. Kusakabe leg.” (EUMJ, 1 male & 1 female; KSCJ, 1 male & 2 females); “Mt. Victoria/ (Natmataung N. P.)/ alt. 1500–2750m/ Kanpelet side”, “Chin Sta., Myanmar/ 21–24. V. 2002/ Y. Kusakabe leg.” (EUMJ, 1 male; KSCJ, 1 male & 1 female); “Mt. Victoria/ alt. 1500–2000 m/ Mindat side”, “Chin Sta., Myanmar/ 13–14.VI.2002/ Y. Kusakabe leg.”, (KSCJ, 1 female). **Thailand**: “Doi Pui Chieng Mai/ N – THAILAND/ 22.V.1986/ leg.” (KSCJ, 1 female); QSBG-2014-0160-0010, Amnat Charoen, Chanuman Dist., Doi Inthanon NP, 18°32'44.4"S, 98°30'53"E, 1376 m, 29.V.-1.VII.2014, Malaise trap, Wichai Srisuka et al. (RGCM, 1 ex.); Same with QSBG….-0008 (RGCM, 1 ex.); Same with QSBG….-0009 + 0011 (QSBG, 3 exs). **China**: S-Yunnan (Xishuangbanna), c. 30 km NW Jinghong, vic. Bameng, 1700–2000 m; Hua Zhuliangzi Mts., 22°08.01'N, 100°31.54'E, 1700–2000 m, 30.V.2008, leg. A. Weigel, sec. forest (RGCM, 1 male).

**Figures 32–35.**
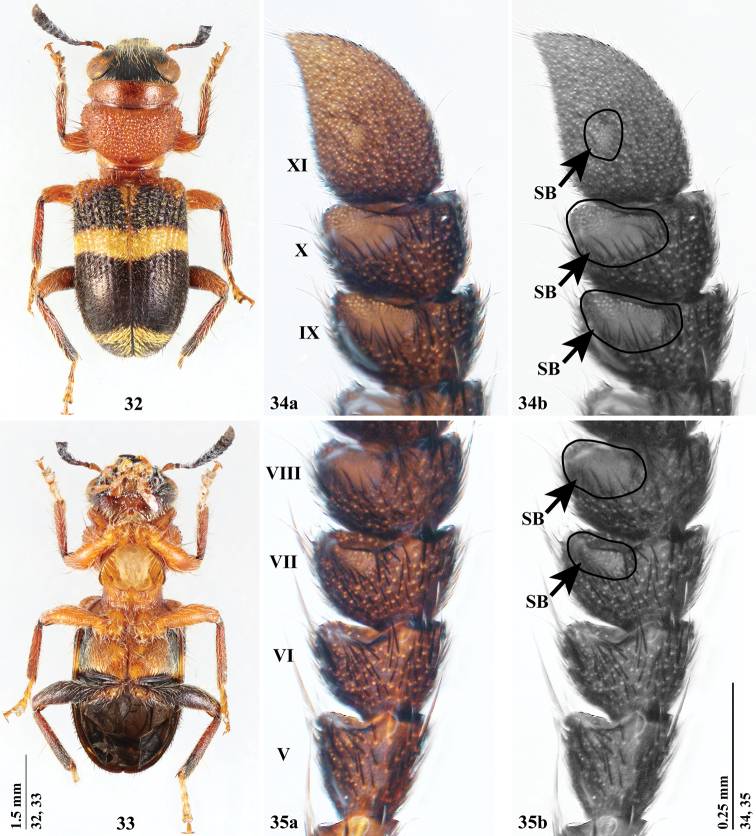
*Hemitrachystubericollis***32, 33** habitus in dorsal (**32**) and ventral (**33**) views **34** male antennomeres IX–XI in ventral view **35** male antennomeres V–VIII in ventral view. Abbreviations: **SB** an area of sensilla basiconica.

#### Diagnosis.

This species is similar to *Tillicerasoror* Schenkling, 1902, but distinguished by the following characteristics: antennomere VI–X gradually broadened; metatibiae apically extended to a broad spine; posterior margin of abdominal ventrite V, with broadly V-shaped emargination; phallobase notched at apical 1/5 of total length of tegmen (vs. *T.soror*: antennomere VIII–IX gradually broadened; hind tibiae not prominent at apex; posterior margin of abdominal sternite V broadly U-shaped and truncate; phallobase deeply notched at apical 1/3).

#### Description.

**Male.** Head, antennomeres IV–XI, base of pronotum and legs brownish black; antennomeres I–III and pronotum reddish. Elytra black except for reddish area near basal suture and yellowish area at basal 1/4 near lateral margin. Head at apex and legs covered with white setae; basal 1/2 of head and pronotum with mingled white and black setae; elytra densely vested with yellowish setae obliquely at suture of basal 1/3 and transversally at lateral margin basal 1/4 and apical 1/4, the remainder vested with black setae (Fig. 10). This species has two color patterns: the first, antennomeres IV–XI, apex of pronotum, elytra except for area covered with yellowish setae, and legs black (Fig. 11); the second, head black; apical 1/4 of pronotum brownish black; elytral setae white and black (Fig. 12).

Head including eyes as wide as pronotum; labrum incised at middle; maxillary terminal palpomeres digitiform; labial terminal palpomeres widely triangular; postgular plate narrow. Antennomere I claviform; II compact; III twice as long as II; IV–X triangular, becoming gradually widened; V–IX (Fig. 30) with an area vested with sensilla basiconica; X (Fig. 29) with two areas vested with sensilla basiconica at apical margin in ventral view; XI (Fig. 28) with small pit-like sensillum in male.

Pronotum slightly longer than wide, widest at middle, with fine dense punctures. Mesoventrite with short anterior process.

Elytra oblong, parallel sided, covered with ten striae; striae I and II absent before basal oblique yellow fascia; III–V or VII extending after middle, sometimes absent before basal oblique yellow fascia; VI or VIII–X rudimentary.

Profemora stouter than meso- and metafemora; punctation of meso- and metafemora denser than that of profemora. Tibiae short prominent at apex, with distinct longitudinal carina on dorsal and ventral surfaces; tibial spur formula 1–2–0; tarsal pulvillar formula 4–4–2; pro- and mesotarsomeres I and II with large lobed pulvilli; III and IV with large bilobed pulvilli; metatarsomeres I and II without pulvilli; III and IV with large bilobed pulvilli; claws with basal denticles.

**Figures 36–40.**
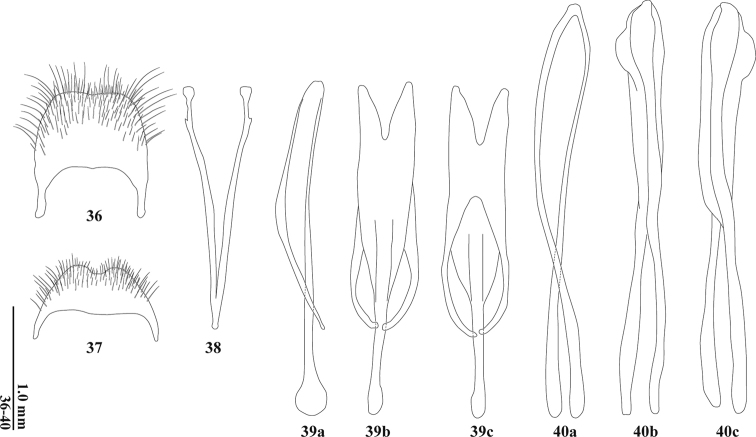
Male terminal parts and aedeagus of *Tilliceracallosa***36** pygidium **37** ventrite VI **38** spicular fork **39** tegmen in lateral (a), ventral (b) and dorsal (c) views **40** median lobe in lateral (a), ventral (b), and dorsal (c) views.

Abdominal ventrite V deeply emarginated at apical margin. Pygidium (Fig. 46) narrowly emarginated at apical margin; ventrite VI (Fig. 47) almost transverse at apical margin; spicular fork long (Fig. 48), without intraspicular plate.

Tegmen (Fig. 49a–c) with dorsal and ventral sinus at apical 1/5; parameroid lobes tapered posteriorly at apex in lateral view; tegminal arms short, extending from middle to basal 1/4 of total length. Median lobe shorter than tegmen; plates with rows of denticles from apical 1/5 to 2/5 of total length on dorsal and ventral sides (Fig. 50a–c).

**Female.** Similar to male but distinguished by antennomere XI without pit-like sensillum, tibial spur formula 1–2–2 and apex of metatibiae not extended to a broad spine.

#### Measurements and ratios.

**Male** (*N* = 4). BL 6.55–8.30 (7.14) mm; PL 1.75–2.30 (1.99) mm; PW 1.55–2.10 (1.78) mm; EL 4.60–6.00 (5.15) mm; EW 2.10–2.65 (2.29) mm; EyW 0.40–0.55 (0.49) mm; EyD 0.55–0.95 (0.76) mm; PL/PW 1.10–1.14 (1.12); EL/EW 2.09–2.36 (2.25); EL/PL 2.24–2.81 (2.60); EW/PW 1.22–1.35 (1.29); EyD/EyW 1.38–1.73 (1.56). **Female** (*N* = 4). BL 8.00–8.90 (8.49) mm; PL 2.20–2.50 (2.36) mm; PW 2.00–2.25 (2.10) mm; EL 5.80–6.50 (6.13) mm; EW 2.50–2.75 (2.65) mm; EyW 0.50–0.60 (0.54) mm; EyD 0.95–1.05 (1.00) mm; PL/PW 1.07–1.20 (1.13); EL/EW 2.23–2.36 (2.31); EL/PL 2.42–2.77 (2.59); EW/PW 1.22–1.31 (1.26); EyD/EyW 1.73–2.00 (1.87).

#### Etymology.

This specific name is derived from the Latin *spinosa* (spine), referring to metatibiae apically extended to a broad spine.

**Figures 41–50.**
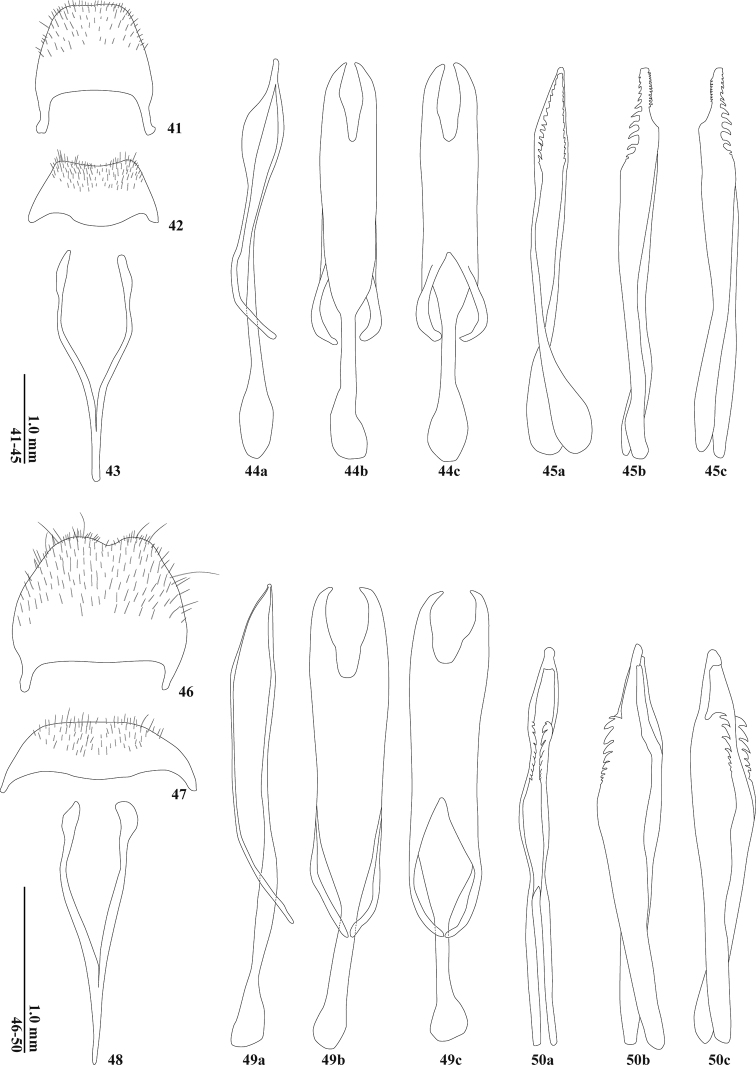
Male terminal parts and aedeagus **41–45***Tillicerafortis* sp. nov. **46–50***T.spinosa* sp. nov. **41, 46** pygidium **42, 47** ventrite VI **43, 48** spicular fork **44, 49** tegmen in lateral (a), ventral (b) and dorsal (c) views **45, 50** median lobe in lateral (a), ventral (b), and dorsal (c) views.

#### Distribution.

Laos, Myanmar, Thailand.

### ﻿Key to the species of *Tillicera* Spinola, 1844

Based on Gerstmeier and Bernhard (2010) and Yang et al. (2011).

**Table:** 

1	Elytra with sickle-like, posteriorly open, semicircle of silvery or golden setae	**2**
–	Elytra without such an arrangement of setae	**8**
2	Brown color of elytral base not reaching the lateral margin of elytra	**3**
–	Brown color of elytral base reaching the lateral margin of elytra	**4**
3	Only antennomere X wider than long or as wide as long; male metatibiae apically not extended to a broad spine (Bhutan, India, Laos, Nepal)	***T.soror* Schenkling, 1902 (Fig. 20)**
–	Antennomeres VIII–X wider than long; male metatibiae apically with a broad spine (Laos, Myanmar, Thailand)	***T.spinosa* sp. nov. (Figs 25–27)**
4	Tarsal pulvillar formula 4–4–3 (Taiwan)	***T.wenii* Yang & Yang, 2011 (Fig. 22)**
–	Tarsal pulvillar formula 4–4–2 or 4-4-4	**5**
5	Pronotum with sparse punctuation (Laos, Thailand)	***T* . *fortis* sp. nov. (Figs 23, 24)**
–	Pronotum with dense punctation	**6**
6	Length to width ratio of terminal antennomere of both sexes > 1.5:1 (China, Laos, Myanmar, Thailand)	***T.sensibilis* Yang & Yang, 2011 (Figs 18, 19)**
–	Length to width ratio of terminal antennomere of both sexes < 1.5:1	**7**
7	Antennomeres IX and X about twice as wide as long (Indonesia, Laos, Malaysia, Thailand, Vietnam)	***T.javana* Spinola, 1844 (Figs 11, 12)**
–	Antennomeres IX and X less than twice wide as long (China, Laos, Myanmar, Thailand, Vietnam)	***T.auratofasciata* (Pic, 1927) (Fig. 1)**
8	Elytral base and humeri red-brown to brown	**9**
–	Elytral base and humeri black, sometimes with brown macula	**13**
9	Pronotum with yellowish fascia at apical margin	**10**
–	Pronotum without yellowish fascia at apical margin	**12**
10	Elytra with large yellowish fascia at apical 1/3, brown at apex (India)	***T.paula* Schenkling, 1908 (Fig. 15)**
–	Elytra with narrow yellowish fascia at apex, black at apex	**11**
11	Elytra largely yellowish red (China, Indonesia, Laos, Myanmar, Thailand)	***T.cleroides* Gorham, 1892 (Figs 5, 6)**
–	Elytra largely black or brownish black (Indonesia, Laos, Malaysia)	***T* . *pseudocleroides* Gerstmeier & Bernhard, 2010 (Figs 16, 17)**
12	Frons about 1.7 times wider than the width of a single eye (Indonesia, Japan, Laos, Myanmar, Taiwan, Thailand)	***T* . *ihlei* Corporaal, 1949 (Figs 9, 10)**
–	Frons about as wide as a single eye (Vietnam)	***T* . *tonkinensis* Gerstmeier & Bernhard, 2010 (Fig. 21)**
13	Elytra with anterior pale fascia at least as broad as the black central part (India)	***T.aurivillosa* Gorham, 1895 (Fig. 2)**
–	Elytra with anterior pale fascia of elytra conspicuously narrower than the black central part	**14**
14	Elytra with a sub-basal hump	**15**
–	Elytra without a sub-basal hump	**16**
15	Transverse fasciae of pattern-forming bright golden setae (China, Laos, Vietnam)	***T* . *michaeli* Gerstmeier & Bernhard, 2010 (Fig. 13)**
–	Transverse fasciae pale, pigmented; setae less brightly golden (India, Vietnam)	***T* . *callosa* Gerstmeier & Bernhard, 2010 (Fig. 4)**
16	Elytral punctation large at base; the space between each puncture within the same interval row narrower than diameter of a single puncture	**17**
–	Elytral punctuation small at base; the space between each puncture within the same interval row larger than or same as diameter of a single puncture (Malaysia)	***T.obscura* Gerstmeier & Bernhard, 2010 (Fig. 14)**
17	Head unicolored black; antennae gradually widening from antennomere V; elytra with longitudinal rows of tubercles on basal intervals (Indonesia, Malaysia, Vietnam)	***T.hirsuta* (Pic, 1926) (Figs 7, 8)**
–	Head reddish brown posteriorly; antennae gradually widening from antennomere VI; elytra without longitudinal rows of tubercles on basal intervals (Cambodia, China, Laos, Myanmar, Thailand, Vietnam)	***T.bibalteata* Gorham, 1892 (Fig. 3)**

## Supplementary Material

XML Treatment for Tillicera

XML Treatment for Tillicera callosa

XML Treatment for Tillicera ihlei

XML Treatment for Tillicera javana

XML Treatment for Tillicera pseudocleroides

XML Treatment for Tillicera soror

XML Treatment for Tillicera tonkinensis

XML Treatment for Tillicera fortis

XML Treatment for Tillicera spinosa
